# Lived experiences of the diagnostic assessment process for fetal alcohol spectrum disorder: A systematic review of qualitative evidence

**DOI:** 10.1111/acer.15097

**Published:** 2023-05-12

**Authors:** Nicole Hayes, Kerryn Bagley, Nicole Hewlett, Elizabeth J. Elliott, Carmela F. Pestell, Matthew J. Gullo, Zachary Munn, Philippa Middleton, Prue Walker, Haydn Till, Dianne C. Shanley, Sophia L. Young, Nirosha Boaden, Delyse Hutchinson, Natalie R. Kippin, Amy Finlay‐Jones, Rowena Friend, Doug Shelton, Alison Crichton, Natasha Reid

**Affiliations:** ^1^ Child Health Research Centre The University of Queensland South Brisbane Queensland Australia; ^2^ Australian Research Council Centre of Excellence for the Digital Child Queensland University of Technology Brisbane Queensland Australia; ^3^ La Trobe Rural Health School La Trobe University Bendigo Victoria Australia; ^4^ Living with Disability Research Centre La Trobe University Melbourne Victoria Australia; ^5^ First Nations Cancer and Wellbeing Research Team The University of Queensland Herston Queensland Australia; ^6^ Faculty of Medicine and Health, Specialty of Child and Adolescent Health The University of Sydney Sydney New South Wales Australia; ^7^ The Sydney Children's Hospitals Network Sydney New South Wales Australia; ^8^ School of Psychological Science University of Western Australia Perth Western Australia Australia; ^9^ School of Applied Psychology Griffith University Mount Gravatt Queensland Australia; ^10^ Health Evidence Synthesis, Recommendations and Impact, School of Public Health, Faculty of Health and Medical Sciences The University of Adelaide Adelaide South Australia Australia; ^11^ South Australian Health and Medical Research Institute Adelaide South Australia Australia; ^12^ The University of Adelaide Adelaide South Australia Australia; ^13^ Victorian Fetal Alcohol Service Monash Children's Hospital Clayton Victoria Australia; ^14^ Australian Childhood Foundation Abbotsford Victoria Australia; ^15^ Child Development Service Gold Coast Hospital and Health Service Southport Queensland Australia; ^16^ School of Applied Psychology Griffith University Gold Coast Queensland Australia; ^17^ Menzies Health Institute of Queensland Griffith University Gold Coast Queensland Australia; ^18^ School of Social Sciences, Faculty of Social Work The University of New South Wales Sydney New South Wales Australia; ^19^ Centre for Social and Early Emotional Development, School of Psychology, Faculty of Health Deakin University Geelong Victoria Australia; ^20^ National Drug and Alcohol Research Centre The University New South Wales Sydney New South Wales Australia; ^21^ Centre for Adolescent Health Murdoch Children's Research Institute, Royal Children's Hospital Melbourne Victoria Australia; ^22^ Department of Paediatrics University of Melbourne, Royal Children's Hospital Melbourne Victoria Australia; ^23^ Curtin School of Allied Health Curtin University Western Australia Bentley Australia; ^24^ Telethon Kids Institute Nedlands Western Australia Australia; ^25^ School of Population Health Curtin University Bentley Western Australia Australia; ^26^ Medical School University of Western Australia Crawley Western Australia Australia; ^27^ Patches Assessment Service Darwin Northern Territory Australia; ^28^ Faculty of Health Charles Darwin University Darwin Northern Territory Australia; ^29^ School of Medicine and Dentistry Griffith University Gold Coast Queensland Australia; ^30^ Community Child Health Gold Coast Hospital and Health Service Southport Queensland Australia; ^31^ Department of Paediatrics Monash University Melbourne Victoria Australia

**Keywords:** clinical guidelines, diagnosis, fetal alcohol spectrum disorder, lived experience, prenatal alcohol exposure, qualitative synthesis

## Abstract

Early assessment and diagnosis of FASD are crucial in providing therapeutic interventions that aim to enhance meaningful participation and quality of life for individuals and their families, while reducing psychosocial difficulties that may arise during adolescence and adulthood. Individuals with lived experience of FASD have expertise based on their own lives and family needs. Their insights into the assessment and diagnostic process are valuable for improving service delivery and informing the provision of meaningful, person‐ and family‐centered care. To date, reviews have focused broadly on the experiences of living with FASD. The aim of this systematic review is to synthesize qualitative evidence on the lived experiences of the diagnostic assessment process for FASD. Six electronic databases, including PubMed, the Cochrane Library, CINAH, EMBASE, PsycINFO, and Web of Science Core Collection were searched from inception until February 2021, and updated in December 2022. A manual search of reference lists of included studies identified additional studies for inclusion. The quality of included studies was assessed using the Critical Appraisal Skills Program Checklist for Qualitative Studies. Data from included studies were synthesized using a thematic analysis approach. GRADE‐CERQual was used to assess confidence in the review findings. Ten studies met the selection criteria for inclusion in the review. Thematic analysis identified 10 first‐level themes relating to four over‐arching topics: (1) pre‐assessment concerns and challenges, (2) the diagnostic assessment process, (3) receipt of the diagnosis, and (4) post‐assessment adaptations and needs. GRADE‐CERQual confidence ratings for each of the review themes were moderate to high. The findings from this review have implications for referral pathways, client‐centered assessment processes, and post‐diagnostic recommendations and support.

## Graphical abstract

This systematic review synthesised qualitative evidence on the lived experiences of the diagnostic assessment process for FASD. The review identified 10 studies addressing 10 themes with four key domains relating to (1) pre‐assessment concerns and challenges, (2) the diagnostic assessment process, (3) receiving of the diagnosis, and (4) post‐assessment adaptions and needs. The review findings have implications for referral pathways, client‐centred assessment processes and post‐diagnostic recommendations and support.

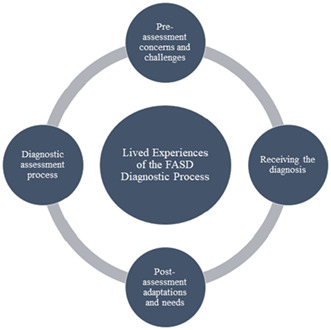

## INTRODUCTION

Fetal alcohol spectrum disorder (FASD) is a neurodevelopmental disability associated with prenatal alcohol exposure (PAE). It is characterized by a broad range of lifelong impairments in cognition, behavior, and learning and can present with or without physical anomalies (Kodituwakku & Kodituwakku, 2014). Many individuals with FASD experience challenges at home, school, work, and in the community (Rangmar et al., 2015; Skorka et al., 2020). Early assessment for and diagnosis of FASD is vital to the provision of therapeutic interventions aimed at improving meaningful participation and quality of life for individuals and their families, and minimizing psychosocial challenges that can occur in adolescence and adulthood (Olson et al., 2007; Streissguth et al., 2004).

Historically, FASD has been underrecognized and in many countries diagnostic services are limited (Clarren et al., 2011; Popova et al., 2020). The assessment for FASD is a comprehensive process, requiring multidisciplinary input. Service‐level barriers to early identification and the diagnosis of FASD include a lack of knowledge of FASD in the health sector and broader community, limited training opportunities for health professionals, and confusion about the diagnostic criteria and referral pathways (Petrenko et al., 2014; Reid et al., 2020). Moreover, information about PAE is not always routinely collected and documented in maternal or child developmental assessments (Doherty et al., 2019) and thus may not be considered a possible contributor to neurodevelopmental challenges. Some health professionals also report a reluctance to diagnose FASD due to concerns about stigmatizing families (Howlett et al., 2019). As a result, delayed diagnosis, misdiagnosis, and inadequate support and treatment are common (Chasnoff et al., 2015; Petrenko et al., 2014).

Although it is important to understand and address system and service‐level barriers to the timely and accurate diagnosis of FASD, client and caregiver perspectives are also vital to improving service delivery. People with lived experience of FASD are experts on their own lives and family needs, and their perspectives on the assessment and diagnostic journey are important to informing the provision of meaningful, person‐, and family‐centerd care (Evans et al., 2022). Evidence across various health settings has shown that incorporating lived experiences in health service decision making leads to responsive and accessible services and can improve quality of care outcomes (Bombard et al., 2018; Crawford et al., 2002). Although several reviews have examined individuals' and caregivers' experiences of living with FASD (e.g., Domeij et al., 2018; Flannigan et al., 2021; Skorka et al., 2020), none have systematically reviewed the evidence of lived experiences of the FASD diagnostic process.

The aim of this systematic review was to synthesize published literature on the lived experiences of individuals and caregivers who have undergone a diagnostic assessment for FASD. We used GRADE‐CERQual, a systematic and transparent approach to assessing the degree of confidence that can be placed on the results of a qualitative evidence synthesis (Lewin et al., 2015). This review will inform the Australian Government‐funded revision and update of the Australian Guide to the Diagnosis of FASD. The importance of representation of lived experiences is well‐recognized in international frameworks on quality clinical guideline development (Institute of Medicine, 2011; National Health and Medical Research Council, 2022). This understanding of lived experience helps to ensure that the needs of individuals and caregivers who will be most affected by the diagnosis are adequately addressed.

## METHOD

### Protocol and registration

The review protocol was registered with the International Prospective Register of Systematic Reviews (PROSPERO Reference: CRD42021230542) and reported according to the Preferred Reporting Items for Systematic Reviews and Meta‐Analysis (PRISMA; Page et al., 2021) and the Enhancing Transparency in Reporting the Synthesis of Qualitative Research guidelines (ENTREQ; Tong et al., 2012).

### Search strategy

A systematic literature search was performed by one author (NHa) using six electronic bibliographic databases: PubMed, the Cochrane Library, CINAHL, EMBASE, PsycINFO, and Web of Science Core Collection from inception until February 2021 (updated December 2022). Table 1 summarizes the search terms used across the databases.

**TABLE 1:** Search terms used for the literature search.

Search topics	Keywords
Fetal alcohol spectrum disorder	“prenatal alcohol” OR “alcohol exposed” OR “fetal alcohol” OR “foetal alcohol” OR “fetal alcohol spectrum disorder” OR “foetal alcohol spectrum disorder” OR “fetal alcohol syndrome” OR “foetal alcohol syndrome” OR “static encephalopathy” OR “alcohol‐related birth defect*” OR “alcohol‐related neurodevelopmental disorder” OR (“neurobehav* disorder” AND “alcohol exposed”) OR (neurobehav* disorder” AND prenatal alcohol”)
	AND
Lived experience	lived OR living OR care* OR caring OR raise* OR parent* OR experienc* OR perspective* OR challeng* OR barrier* OR impact* OR need* OR perceive Or “daily life” OR “daily liv*”
	AND
Diagnostic assessment	assess* OR diagnos* OR evaluat* OR screen* OR clinic OR service OR system OR “health care” OR support

Retrieved references were imported to an Endnote library, duplicate records were removed, and references were uploaded to Covidence systematic review software (Veritas Health Innovation, 2021). Title and abstracts were independently screened for eligibility against inclusion and exclusion criteria by two authors (NHa, SY). Full‐text publications of relevant references were retrieved and independently assessed by the two authors. Discrepancies were resolved via discussion with a third author (NR). Manual screening of reference lists of retrieved full‐text publications was performed to identify relevant publications not identified by the initial search strategy.

### Study selection criteria

Articles were included if they: (1) were published in English; (2) reported qualitative or mixed methods primary research; (3) reported lived experiences for individuals and/or caregivers of individuals with FASD; and (4) reported at least one theme relating to the experiences of diagnostic assessment for FASD or receipt of a diagnosis of FASD. Articles were excluded if: (1) the study included other participants (i.e., health professionals) and data on individuals or caregivers of individuals with FASD could not be extracted separately; (2) the study reported only on the experiences of living with FASD; and (3) publications were theses, conference proceedings/abstracts, or literature reviews.

### Study quality assessment

The quality of included studies was assessed independently by two authors (NHa and NB) using the Critical Appraisal Skills Programme (CASP) Checklist for Qualitative Studies (Critical Appraisal Skills Programme, 2018). Items are evaluated as “Yes,” “Partial,” “Unsure,” and “No.” Discrepancies in quality appraisal ratings were resolved via discussion with a third author (NR).

### Data extraction and synthesis

One author (NHa) extracted data from each study using a standard form that included article information (author, year, and country), stated aim, study design, study population, and relevant key findings including stated themes and subthemes. Another author (NHe) checked the data extraction.

A thematic approach (Thomas & Harden, 2008) was used to synthesize data. Major themes, subthemes, and participant quotes related to lived experiences of the diagnostic assessment process and receipt of FASD diagnosis were extracted from each article. Inductive analysis was used whereby extracted data were read line‐by‐line and coded, with common emerging codes grouped to form first‐level themes. Themes were then categorized into overarching topics. Thematic coding was undertaken by one author (NHa) then discussed with two other authors (KB and NR). All three authors have experience in qualitative research methods and clinical research in FASD. Additionally, NR is a clinician with experience in the diagnostic assessment of FASD. KB has clinical experience in intervention and support for individuals with FASD and their families.

The Grading of Recommendations, Assessment, Development, and Evaluation (GRADE)‐Confidence in the Evidence from Reviews of Qualitative Research (CERQual) (Lewin et al., 2015) was used to assess confidence in the findings. CERQual provides an assessment of the extent to which each review theme is a reasonable representation of the phenomenon of interest. The assessment includes four components: methodological limitations, relevance, coherence, and adequacy of the data, with concerns on each component rated as no/very minor, minor, moderate, or serious. Overall confidence has four levels: high, moderate, low, or very low. Review themes start at “high confidence” and are rated down one or more levels if there are concerns related to any components. Two authors (NHa and KB) completed the CERQual Ratings collaboratively through discussion. Table S1 provides explanations for each rating.

## RESULTS

### Study selection and characteristics

**FIGURE 1:**
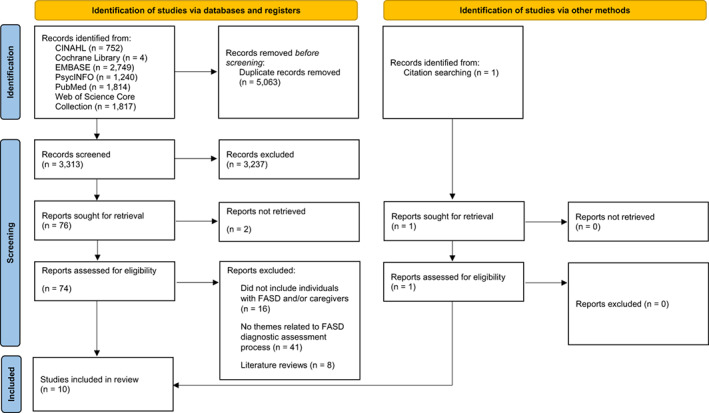
PRISMA flow diagram for the selection of studies.

The database search identified 8376 records. Following duplicate removal, 3313 records were screened by title and abstract and 74 were assessed at the full‐text level. Ten studies met the inclusion criteria and were included in the review (Figure 1). Study characteristics of the 10 included studies are presented in Table 2. Studies were from Canada (*n* = 4), Australia (*n* = 3), the United States (*n* = 1), New Zealand (*n* = 1), and UK (*n* = 1). Nine studies described the experiences of caregivers of children and young people, with most studies (*n* = 7) including a mixed sample of foster and adoptive caregivers, biological parents, grandparents, and other relatives, of which one study reported including both male and female caregivers. Two studies described the experiences of biological mothers, and one study described the experiences of female Aboriginal Australian caregivers, including biological mothers and grandmothers. One study described the experiences of adults diagnosed with FASD.

**TABLE 2:** Study characteristics of included studies.

Author(s) (year), country	Research aim	Participants	Data collection and analysis methods	Relevant study themes^a^
Chamberlain et al. (2017) Australia	Explore the lived experience of the diagnostic process for caregivers of children with FASD	10 primary caregivers (4 foster parents; 1 adoptive parent; 5 legal guardians) of children (aged 6 to 12 years) who received a FASD diagnosis from a multidisciplinary specialist diagnostic service	Semistructured interviews; phenomenological approach and thematic analysis	Caregivers' aspirations and actions to enhance their child's future Increased caregiver uncertainty Caregiver knowledge and understanding of FASD Lack of societal knowledge and recognition of FASD Assessment provided validation and understanding Process of diagnosis as empowering
Doak et al. (2019) Australia	Explore the lived experiences of Australian caregivers who received a FASD diagnosis for a child in their care	7 caregivers (3 biological mothers, 1 biological father, 1 maternal grandmother and 2 paternal grandmothers) of children (aged 3 to 13 years) who attended a specialist multidisciplinary FASD clinic and received a FASD diagnosis (*n* = 6) or an at risk of FASD diagnosis (*n* = 1)	Semi‐structured interviews; phenomenological approach and thematic analysis	Receiving a FASD diagnosis had a positive impact Caregivers' evaluation of assessment process Positive support services relative to FASD Ongoing difficulties regardless of diagnosis Need for societal knowledge of FASD
Duquette and Stodel (2005) Canada	Gain an understanding of the school experiences of children with FASD and to identify the elements of a successful school experience from the perspective of children with FASD and their parents	11 adoptive parents (middle class from the cultural majority) of an individual (aged ≥9 years) with FASD	Questionnaire with open‐ended questions and semi‐structured interviews; Grounded theory, qualitative analysis	Obtaining a diagnosis
Hamilton et al. (2020) Australia	Explore experiences of FASD diagnostic assessment among caregivers of detained young people	15 caregivers (2 couples, 6 mothers, 5 grandmothers; 10 Aboriginal and 5 non‐Aboriginal caregivers) of young person (aged 10 to 17 years) who received a multidisciplinary FASD diagnostic assessment as part of a larger FASD prevalence study among sentenced, detained youth	Yarning; ontological approach with interpretivist lens and thematic network analysis	Conceptualisation of diagnosis Diagnostic reports and resources Post‐diagnosis support
Petrenko et al. (2014) U.S.	Examine system‐level barriers that contribute to the development of secondary conditions in FASD	25 parents (1 biological mother, 24 adoptive/foster parents) of individuals (aged 3 to 33 years) with FASD	Individual interview or focus groups; Phenomenological approach using framework analysis	Delayed diagnosis Qualifying for services Availability of services
Salmon (2008) New Zealand	Describe the lived experiences of biological mothers, from pregnancy onwards, of a child/ren diagnosed with FASD	8 biological mothers who had nurtured or were still living with their offspring (aged 8.5 to 30 years) diagnosed with FASD	Unstructured individual interviews; constant comparative method, validity: triangulation	Medical and health professionals abandon the mothers
Sanders and Buck (2010) Canada	Investigate parents' experiences raising children with FASD	11 caregivers (3 biological, 7 adoptive, 1 foster) of individuals (aged 5 to 21 years) with FASD	Unstructured individual interviews; thematic analysis	Something's not right Receiving a diagnosis
Temple et al. (2021) Canada	To understand how receiving a diagnosis of FASD in adulthood might influence outcomes; and investigate the long‐term outcomes for individuals diagnosed with FASD after 18 years of age	20 adults (aged 18 to 45 years) who were referred to a diagnostic clinic and received an FASD after 18 years of age	Survey (open‐ended questions); qualitative analysis	Reactions and thoughts about receiving an FASD diagnosis
Thomas and Mukherjee (2019) U.K.	Explore experiences of biological mothers following a diagnosis of FASD in their children	5 biological mothers of children with FASD (age from 10 to 29 years)	Individual semi‐structured interviews; Interpretative phenomenological analytical approach	Life is a series of battles
Watson et al. (2013) Canada	Examine the experience of raising a child with a disability	31 parents (biological parents, foster parents, adoptive parents, step‐parents and custodial grandparents) of children with FASD (aged from 1 to 36 years)	Semi‐structured interviews (individually or couples), follow‐up questions by email or telephone; interpretative phenomenological analysis	The FASD diagnostic process A family's need for a label

^a^ Hamilton et al. (2020) presented results as vignettes. Main themes presented in this table have been summarized by the review authors drawing on information presented in the vignettes.

### Quality of the included studies

The quality appraisal of the 10 included studies is presented in Table 3. All studies had clear aims and appropriate qualitative methodologies. Most clearly reported their sampling and data collection methods. Only three of the 10 studies provided information about the role of the researcher. Most studies confirmed ethics approval and procedures to obtain participant informed consent. Most studies clearly described the data analysis, although two did not specify the qualitative data analysis method used, and one study provided limited information about the data analytic process. The findings of all studies were clearly presented and contributed to developing an understanding of lived experiences of the diagnostic process for FASD.

**TABLE 3:** Quality assessment of included studies.

CASP qualitative checklist	Chamberlain et al. (2017)	Doak et al. (2019)	Duquette and Stodel (2005)	Hamilton et al. (2020)	Petrenko et al. (2014)	Salmon (2008)	Sanders and Buck (2010)	Temple et al. (2021)	Thomas and Mukherjee (2019)	Watson et al. (2013)
Clear statement of aim	Yes	Yes	Yes	Yes	Yes	Yes	Yes	Yes	Yes	Yes
Qualitative methodology	Yes	Yes	Yes	Yes	Yes	Yes	Yes	Yes	Yes	Yes
Research design	Yes	Yes	Yes	Yes	Yes	Yes	Yes	Yes	Yes	Yes
Sampling	Yes	Yes	Yes	Yes	Yes	Yes	Yes	Yes	Yes	Yes
Data collection	Yes	Yes	Yes	Yes	Yes	Partial	Unclear	Yes	Yes	Yes
Researcher reflexivity	Partial	Partial	No	Partial	No	No	No	No	No	No
Ethical consideration	Yes	Yes	Unclear	Yes	Yes	Yes	Partial	Yes	Yes	Yes
Data analysis	Yes	Yes	Unclear	Yes	Yes	Yes	Yes	Unclear	Partial	Yes
Clear statement of findings	Yes	Yes	Yes	Yes	Yes	Yes	Yes	Yes	Yes	Yes
Research value	Yes	Yes	Yes	Yes	Yes	Yes	Yes	Yes	Yes	Yes

### Qualitative synthesis of lived experiences of the FASD diagnostic assessment process

Thematic analysis identified 10 first‐level themes relating to four over‐arching topics: (1) pre‐assessment concerns and challenges, (2) the diagnostic assessment process, (3) receipt of the diagnosis, and (4) post‐assessment adaptations and needs. A summary of these 10 themes and associated CERQual assessments are presented in Table 4.

**TABLE 4:** Review findings and GRADE‐CERQual ratings.

Review findings	GRADE‐CERQual assessment		
	Contributing studies	Confidence rating	Explanation
**Pre‐assessment concerns and challenges**			
*Caregiver recognition and help‐seeking for child's challenges:* Caregivers reported recognizing behavioral challenges that prompted them to seek help. Challenges included oppositional defiance, aggression and severe impulsivity	3 studies (Chamberlain et al., 2017; Salmon, 2008; Sanders & Buck, 2010)	Moderate	Rated down one level due to minor concerns related to methodology limitations of the contributing studies, minor concerns about coherence across studies, and minor concerns related to assessment of adequacy due to small sample size and limited data
*Dismissal of caregiver's concerns by health professionals:* Caregivers perceived previous health services to be unhelpful and, in some cases, negative, including not feeling listened to and having their concerns dismissed	3 studies (Chamberlain et al., 2017; Salmon, 2008; Sanders & Buck, 2010)	High	Did not rate down. Minor concerns related to methodological limitations, although no concerns related to relevance, coherence or adequacy
*FASD not considered or acknowledged:* Caregivers reported that FASD was not considered as a possible diagnostic outcome, even when caregivers raised the topic of PAE/FASD with health professionals	7 studies (Doak et al., 2019; Duquette & Stodel, 2005; Petrenko et al., 2014; Salmon, 2008; Sanders & Buck, 2010; Thomas & Mukherjee, 2019; Watson et al., 2013)	High	Did not rate down. Minor concerns related to methodological limitations, although no concerns related to relevance, coherence or adequacy
**Diagnostic assessment process**			
*Limited availability of diagnostic assessment services:* Caregiver's described frustrations with accessing assessment services for FASD due to the limited number of and long waitlists when services were available	3 studies (Petrenko et al., 2014; Thomas & Mukherjee, 2019; Watson et al., 2013)	Moderate	Rated down one level due to minor concerns related to methodology limitations, minor concerns about coherence of findings across studies, and minor concerns related to adequacy due to limited data
*A safe and supportive environment without judgment is validating and empowering:* Caregivers reported positive experiences with high levels of satisfaction and feelings of empowerment when attending a specialist FASD service. This was attributed to welcoming, supportive interactions with clinic staff who were helpful, reassuring, and respectful without being judgmental or stigmatizing	2 studies (Chamberlain et al., 2017; Doak et al., 2019)	Moderate	Rated down one level due to very minor concerns related to methodological limitations, and minor concerns related to relevance of the samples and adequacy of the data, although no concerns related to coherence
*Strengths‐based diagnostic reports are a valuable resource:* The diagnostic reports were noted as a valuable resource by caregivers to help them and others working with their child to understand strengths and areas of vulnerability. Caregivers reported intentions to share reports with future health professionals, social services, youth justice personnel, and school staff	3 studies (Chamberlain et al., 2017; Doak et al., 2019; Hamilton et al., 2020)	High	Did not rate down. Very minor concerns related to methodological limitations, although no concerns related to relevance, coherence or adequacy
**Receiving the diagnosis**			
*Mixed emotions and improved insight:* While mixed feelings were experienced when receiving a FASD diagnosis, including a sense of relief, hope and confidence, as well as grief, hopelessness, guilt and shame, the diagnosis also provided improved understanding and insight	8 studies (Chamberlain et al., 2017; Doak et al., 2019; Duquette & Stodel, 2005; Hamilton et al., 2020; Sanders & Buck, 2010; Temple et al., 2021; Thomas & Mukherjee, 2019; Watson et al., 2013)	High	Did not rate down. Minor concerns related to methodological limitations, although no concerns related to relevance, coherence or adequacy
*Means to receive appropriate and tailored support:* Adult individuals and caregivers perceived the benefits of the diagnosis as a means to access appropriate support and services tailored to their own/their child's needs	6 studies (Chamberlain et al., 2017; Doak et al., 2019; Duquette & Stodel, 2005; Hamilton et al., 2020; Temple et al., 2021; Watson et al., 2013)	High	Did not rate down. Minor concerns related to methodological limitations, although no concerns related to relevance, coherence or adequacy
**Post‐assessment adaptations and needs**			
*Aspirations and apprehensions about the future*: Caregivers recognized their child's strengths, and with appropriate support, expressed aspirations for a fulling life for their child. At the same time, caregivers expressed apprehension about their child's future, acknowledging uncertainties related to ongoing difficulties and complexities of secondary conditions	2 studies (Chamberlain et al., 2017; Doak et al., 2019)	Moderate	Rated down one level due to minor concerns related to methodological limitations and adequacy. No concerns related to relevance or coherence
*Accessing supports and services:* Caregivers described various service‐ and family‐level barriers in accessing post‐assessment support, including a lack of local FASD‐specific services and providers knowledgeable in FASD and long waitlists for allied health services, as well as family and work commitments, financial strain and family stress	5 studies (Chamberlain et al., 2017; Doak et al., 2019; Hamilton et al., 2020; Petrenko et al., 2014; Watson et al., 2013)	High	Did not rate down. Very minor concerns related to methodological limitations and minor concerns related to coherence, although no concerns related to relevance or adequacy

#### Pre‐assessment concerns and challenges

##### Theme: “Somethings not right”^1^: Caregiver recognition and help‐seeking for child's challenges

In three studies (Chamberlain et al., 2017; Salmon, 2008; Sanders & Buck, 2010), the assessment journey typically commenced when caregivers recognized behavioral challenges that prompted them to seek help (*GRADE CERQual confidence level: Moderate*). These challenges included oppositional defiance, aggressive behavior, and severe impulsivity, that for some children, was out of character. Caregivers also reflected on sleep problems, feeding difficulties and delayed developmental milestones when their child was younger. Chamberlain et al. (2017) reported that caregivers' parenting knowledge and experiences assisted them to recognize when their child's behavior was of concern to them.

Caregivers were proactive in contacting various health professionals and services, such as speech pathology, occupational therapy and educational psychology and information seeking through online researching, reading books, and other materials (Chamberlain et al., 2017; Sanders & Buck, 2010). Caregiver also reported implementing practical support strategies, such as engaging their child in dancing classes to improve coordination skills (Chamberlain et al., 2017).

##### Theme: “Nothing to worry about”^1^: Dismissal of caregiver's concerns by health professionals

In three studies (Chamberlain et al., 2017; Salmon, 2008; Sanders & Buck, 2010), caregivers reported accessing numerous services for their child's behavioral concerns but perceived these to be unhelpful and in some cases negative, including not feeling listened to and having their concerns dismissed by health professionals (GRADE *CERQual confidence level: High*). In one study (Salmon, 2008), caregivers reported being viewed as a “neurotic” or “hypochondriac” by health professionals and one caregiver said their adolescent's behavior was labeled as “attention‐seeking” (ps. e201‐2). Salmon (2008) also reported that many biological mothers felt unsupported and “left to it, without sufficient information or explanation” for their child's behavioral challenges (p. e201). In one study (Chamberlain et al., 2017), caregivers reported feeling judged about their parenting style and said their child's difficulties were often attributed to family or environmental factors.

##### Theme: “Not on the radar”^1^: FASD not considered or acknowledged

In seven studies (Doak et al., 2019; Duquette & Stodel, 2005; Petrenko et al., 2014; Salmon, 2008; Sanders & Buck, 2010; Thomas & Mukherjee, 2019; Watson et al., 2013), caregivers reported that FASD was not considered as a possible diagnostic outcome, even when caregivers raised the topic of PAE/FASD with health professionals (*GRADE CERQual confidence level: High*). In two studies (Salmon, 2008; Thomas & Mukherjee, 2019), biological mothers who disclosed alcohol consumption during pregnancy in the context of seeking a diagnosis, reported that health professionals refused to acknowledge FASD and described the label as associated with guilt and stigma for families. Caregivers also reported misconceptions among health professionals about timing of PAE and FASD, with one biological mother told that FASD “was not possible” due to drinking “too late in the game” (Watson et al., 2013, p. 108).

There was frustration among caregivers at the lack of FASD knowledge among health professionals when they attempted to seek a diagnosis, with reports that nobody “seemed to be cognizant of fetal alcohol” (Sanders & Buck, 2010, p. 312) or “had ever heard of FASD” (Salmon, 2008, p. e200). Some caregivers reported that when children did not have any discernible facial features, it was difficult to obtain a FASD diagnosis (Petrenko et al., 2014; Watson et al., 2013). This led to delays in receiving an accurate diagnosis and meant that children were initially assessed for alternative conditions. Doak et al. (2019) reported that nine of the 10 children included in their study had previously received another developmental‐behavioral diagnosis, with caregivers in other studies similarly reporting prior diagnoses of autism, ADHD, learning disabilities or other mental health conditions (Duquette & Stodel, 2005; Petrenko et al., 2014; Sanders & Buck, 2010).

There was also a lack of prior knowledge of FASD among some caregivers. Doak et al. (2019) reported that more than half of caregivers said they were unaware of FASD until it was raised by a health professional, and one caregiver attributed their child's challenges to depression due to trauma. Some caregivers reported becoming aware of FASD as a potential diagnosis only when hearing others' stories, usually from television programs or movies, and recognizing similar challenges for their child (Duquette & Stodel, 2005; Petrenko et al., 2014). Sanders and Buck (2010) reported that while some nonbiological caregivers knew that alcohol was consumed during pregnancy, they were uninformed of the potential effects on their child's cognitive, learning, and behavioral functioning. In contrast, Chamberlain et al. (2017) reported that most caregivers had some prior understanding of FASD and believed it underpinned their child's difficulties prior to assessment. One caregiver in the study by Sanders and Buck (2010), who was aware of PAE, suspected FASD and initiated the diagnostic process when her child presented with “extreme behaviors” (p. e312).

#### Diagnostic assessment process

##### Theme: “It shouldn't be this hard”^2^: Limited availability of diagnostic assessment services

Three studies (Petrenko et al., 2014; Thomas & Mukherjee, 2019; Watson et al., 2013) described caregiver's frustrations with accessing assessment services for FASD due to the limited number of providers and long waitlists when services were available (*GRADE CERQual confidence level: Moderate*). Petrenko et al. (2014) noted that participants identified only one provider in their region who had expertise in FASD assessment. In another study (Watson et al., 2013), caregivers reported having to videoconference with doctors and travel long distances to see specialists, while others (Thomas & Mukherjee, 2019) reported limited access to health professionals skilled in FASD diagnoses.

##### Theme: “People listened to me, they listened to the kids”^3^: A safe and supportive environment without judgement is validating and empowering

Two studies (Chamberlain et al., 2017; Doak et al., 2019) reported positive experiences with high levels of satisfaction and feelings of empowerment when attending a specialist FASD service (*GRADE CERQual confidence level: Moderate)*. In both studies, caregivers described welcoming, supportive interactions with clinic staff who were helpful, reassuring, and respectful without being judgmental or stigmatizing. Biological mothers reported feeling accepted and did not feel blame from staff (Doak et al., 2019). Caregivers valued an assessment process that made them feel heard, and allowed for rapport building with both the caregiver and child (Chamberlain et al., 2017). For many caregivers, the assessment process was referred to as a validation of their experiences, often after a long period of searching for answers. Chamberlain et al. (2017) also noted that a multidisciplinary assessment was important for caregivers, enabling them to understand the extent and nature of their child's difficulties.

##### Theme: “Share it with anyone who would listen”^4^: Strengths‐based diagnostic reports are a valuable resource

In three studies (Chamberlain et al., 2017; Doak et al., 2019; Hamilton et al., 2020), the diagnostic reports were noted by caregivers as a valuable resource to help them and others working with their child to understand strengths and areas of vulnerability (*GRADE CERQual confidence level: High*). Caregivers reported intentions to share reports with future health professionals, social services, youth justice personnel, and school staff. The diagnostic reports affirmed the difficulties caregivers experienced and empowered them to better convey their child's needs to other professionals (Chamberlain et al., 2017). Caregivers also reported benefits of a feedback session to schools provided by FASD diagnostic clinic staff in creating an understanding of their child's needs within the education context, enabling accommodations at school to assist their learning (Doak et al., 2019). Hamilton et al. (2020) noted the important differences in understanding diagnostic reports across client populations and demographics, with Aboriginal Australian caregivers describing difficulty engaging with Western medical information and the need for assistance to translate and better understand the language used.

Recommendations for supports were viewed by caregivers as an important aspect of feedback and diagnostic reports. Practical, strength‐based strategies were particularly valued, and Aboriginal Australian caregivers noted that visual strategies were highly beneficial for communicating with their child or young person (Hamilton et al., 2020). However, some caregivers did not know how to implement the strategies, perceiving some recommendations as “unattainable” and beyond the reach of the family (Doak et al., 2019). One caregiver (Hamilton et al., 2020) questioned the relevance of the recommendations in the context of a lack of local services. Some caregivers reported feeling overwhelmed by the end of the feedback session and were not aware that recommendations for ongoing support had been given (Doak et al., 2019).

#### Receiving the diagnosis

##### Theme: “It was really hard at first, but it was a relief to understand”^5^: Mixed emotions and improved insight

Eight studies (Chamberlain et al., 2017; Doak et al., 2019; Duquette & Stodel, 2005; Hamilton et al., 2020; Sanders & Buck, 2010; Temple et al., 2021; Thomas & Mukherjee, 2019; Watson et al., 2013) reported that while mixed feelings were experienced when receiving a FASD diagnosis, including a sense of relief, hope, and confidence, as well as grief, hopelessness, guilt, and shame, the diagnosis also provided improved understanding and insight (*GRADE CERQual confidence level: High*). Adults diagnosed with FASD reported that the diagnosis provided a sense of relief at knowing the reason for the challenges they experienced (Temple et al., 2021). Adults described that the diagnosis allowed them to have fewer negative feelings about themselves, because in the past they had experienced guilt and sometimes blamed themselves for the problems they experienced. Some adults described difficulty in accepting the lifelong nature of the disability and were ambivalent and struggling with what the diagnosis meant for their future (Temple et al., 2021).

In all caregiver groups, caregivers reported relief in receiving a diagnosis of FASD for their child, with a renewed sense of confidence to move forward and advocate for their child's needs (Chamberlain et al., 2017; Doak et al., 2019; Duquette & Stodel, 2005; Hamilton et al., 2020; Sanders & Buck, 2010; Thomas & Mukherjee, 2019). This sense of relief was primarily driven by the long journey in seeking an accurate diagnosis and validation of concerns. The diagnostic clarification and further information about FASD and the impacts of PAE reframed caregivers' expectations and helped them to understand their child's capabilities. Caregivers reported a new understanding of why their child behaved in certain ways, including putting into perspective certain tasks the child struggled with, as well as the reason for underlying academic, emotional, and social difficulties (Doak et al., 2019; Duquette & Stodel, 2005). They stopped blaming their child for causing some of the problems, understanding that behaviors were not intentional. Coupled with this positive reflection on the diagnosis, caregivers also experienced a grieving process and felt overwhelmed. Some caregivers described the “double whammy” of multiple diagnoses that indicated a complex profile for their child (Sanders & Buck, 2010, p. e312). Caregivers expressed feelings of guilt and regret related to mishandling previous behaviors they had considered as defiant and naughty (Chamberlain et al., 2017; Doak et al., 2019). There was an additional expression of grief and guilt by biological mothers, knowing that their alcohol consumption during pregnancy was related to their child's difficulties (Hamilton et al., 2020; Sanders & Buck, 2010; Thomas & Mukherjee, 2019). Some biological caregivers shared concerns regarding their future relationship with their child and how they may come to blame them for their difficulties.

Hamilton et al. (2020) explored responses to the diagnosis among Aboriginal and non‐Aboriginal Australian caregivers. The way in which Aboriginal Australian caregivers discussed alcohol consumption during pregnancy largely reflected the intergenerational trauma and shame experienced by many Aboriginal peoples as a result of colonization and its ongoing impacts. Such experiences of shame underpinned fears of stigma and additional negative labels that could accompany a diagnosis of FASD for Aboriginal Australians. Concerns about the impact of the diagnosis were described in the context of how such a diagnosis would impact on connections to culture and community, for example, their child's participation in cultural ceremonies and involvement in community sports.

##### Theme: “Without the name, there is no place to begin”^6^: Diagnosis is a means to receive appropriate and tailored support

Six studies (Chamberlain et al., 2017; Doak et al., 2019; Duquette & Stodel, 2005; Hamilton et al., 2020; Temple et al., 2021; Watson et al., 2013) reported that adults with FASD and caregivers perceived the benefits of the diagnosis as a means to access appropriate support and services tailored to their own/their child's needs (*GRADE CERQual confidence level: High*). Adults with FASD reported that the diagnosis enabled application for financial and other therapeutic supports, such as disability plans and social services support (Temple et al., 2021). They also spoke of receiving support from family members, employers and other agencies, noting that interactions were now “different in a way that was better for their needs” (Temple et al., 2021, p. 6). For children and young people, caregivers described hopes that their children would now be able to receive more appropriate and individualized support from health professionals and other social services. For school‐aged children, the diagnosis was important in promoting advocacy for and access to additional support within the education system, with some caregivers reporting that educators were more willing to listen to them and make accommodations after their child received a diagnosis (Doak et al., 2019; Duquette & Stodel, 2005). One caregiver of a child currently in youth detention had shared the diagnostic report with a service provider to plan for appropriate health and disability support upon their child's release (Hamilton et al., 2020).

Observable cultural differences in how caregivers envisioned their child's needs being met following the diagnosis were described by Hamilton et al. (2020), with non‐Aboriginal Australian caregivers speaking of hope for more institutional assistance from schools and the healthcare system, and Aboriginal Australian caregivers anticipated their child's support needs would be met within the context of family and community.

#### Post‐assessment adaptations and needs

##### Theme: “That's all I want for them, just to have a nice life”^7^: Aspirations and apprehensions about the future

In two studies (Chamberlain et al., 2017; Doak et al., 2019), caregivers described both aspirations and apprehensions for their child's future following the assessment (*GRADE CERQual confidence level: Moderate*). The diagnosis helped caregivers to acknowledge their child's capabilities, be more empathic and accepting of their child's behavior, and make accommodations to assist their child's success with everyday tasks and routines. With appropriate support from family, friends and services, caregivers expressed aspirations for a fulfilling life for their child. At the same time, caregivers expressed apprehension about their child's future, acknowledging uncertainties related to ongoing difficulties and complexities of secondary conditions. Some caregivers raised concerns about their child's physical safety, ability to learn at school, and future mental health (Doak et al., 2019). Others reported concerns about their child's future independent living skills and their own ability to care for their child in the long term (Chamberlain et al., 2017).

##### Theme: “Our struggle is finding a counsellor who knows enough about FASD to be able to help”^8^: Accessing supports and services

Across five studies (Chamberlain et al., 2017; Doak et al., 2019; Hamilton et al., 2020; Petrenko et al., 2014; Watson et al., 2013), caregivers described service‐ and family‐level barriers to accessing support (*GRADE CERQual confidence level: High*). At the service level, there was a reported lack of local FASD‐specific services and providers knowledgeable about FASD, and long waitlists for allied health services. In the context of the lack of local community services, some caregivers questioned the benefits of diagnosis (Chamberlain et al., 2017; Hamilton et al., 2020). In studies by Petrenko et al. (2014) in the United States and Watson et al. (2013) in Canada, caregivers reported difficulty qualifying for services postdiagnosis, because FASD was not classified as a developmental disability in local education systems and their child did not have a recognized intellectual disability (IQ < 70) or developmental disability (e.g., autism spectrum disorder). At the family level, barriers to accessing services included family and work commitments, financial strain, and family stress (Doak et al., 2019). Some caregivers described feeling stigmatized and isolated from their community, due to a lack of understanding by others about their child's behavior (Doak et al., 2019; Petrenko et al., 2014). This affected families' participation in social activities (e.g., play dates). Across all five studies, caregivers expressed hope and desire for FASD to be more widely recognized so that appropriate programs could be implemented to support individuals across the lifespan.

Despite numerous barriers, some caregivers did report accessing several helpful supports following diagnosis (Doak et al., 2019). These included an instructional book on prosocial behavior, a specialized unit at school, support from the school's guidance officer, medications to manage ADHD and sleep problems, occupational therapy, physical exercise regimens, in‐home counseling, and interventions from a private psychologist. Importantly, caregivers identified benefits of accessing supports to assist them personally, particularly peer support groups and extended family support.

## DISCUSSION

The aim of this systematic review was to synthesize qualitative evidence on the lived experiences of the diagnostic assessment process for FASD. The review identified 10 studies addressing 10 themes within four key domains relating to (1) pre‐assessment concerns and challenges, (2) the diagnostic assessment process, (3) receiving the diagnosis, and (4) post‐assessment adaptations and needs. Confidence ratings for most review themes were high, indicating that these themes are a reasonable representation of people's experiences of the assessment and diagnostic process, although the moderate confidence ratings for some themes reflected concerns about the adequacy of the data and indicate that further research is needed.

Behavioral challenges frequently led caregivers to seek help, particularly impulsive and aggressive behaviors that were difficult to manage. An association between PAE and externalizing behavior problems has been widely reported (Mattson et al., 2011; Tsang et al., 2016) with significant impacts on caregiver stress and family functioning (Bobbitt et al., 2016; Reid & Moritz, 2019). Prior to diagnosis, many caregivers reported that their behavior‐related concerns were dismissed by health professionals or considered part of alternative developmental diagnoses. This is not uncommon, with previous statistics from Canada showing a missed diagnosis rate of 80% for FASD and a misdiagnosis rate of 6.4% (Chasnoff et al., 2015). Early developmental concerns identified by caregivers are often accurate and are later validated by physicians as medically significant (Simon et al., 2022), underscoring the importance of acknowledging the expertise that caregivers bring regarding their child's life. Family‐centered care that values working collaboratively with families has previously been suggested as key to FASD‐informed care delivery (Joly et al., 2022; Reid, Crawford, et al., 2022) and could be considered as a critical approach to the assessment and diagnostic process.

A common observation was that a diagnostic assessment was difficult to access, due primarily to a lack of local services. However, when caregivers could access specialist FASD diagnostic clinics, their experiences were positive and empowering, with feelings of being heard in a nonjudgmental manner, without blame and shame. This appreciation for specialist diagnostic clinics among end‐users may reflect specialist clinicians' awareness, knowledge and experience regarding FASD and their ability to support caregivers through the assessment process. Recent international literature, however, has noted that access to best practice multidisciplinary FASD diagnosis is limited, can be costly, and often unavailable, especially in regional and remote areas (Dugas et al., 2022; Popova et al., 2020). The review findings suggest that clearer referral pathways are needed, in addition to promoting skills in FASD assessment and diagnosis within more general primary healthcare settings.

Consistent with research exploring other developmental and behavioral conditions (Donders, 2020; Evans et al., 2022), the reviewed evidence highlighted the benefits of feedback sessions and reports for clarifying the FASD diagnosis and guiding future strategies and supports. Caregivers particularly valued the focus on the individual's strengths along with understanding areas of vulnerability. Strength‐based approaches to feedback and reporting can provide a more positive and empowering approach to diagnosis by allowing individuals and families to recognize unique strengths, abilities, and interests, and identify the strategies and supports that build on those strengths and positive attributes. This aligns with a growing body of literature in the FASD field highlighting the importance of strengths‐based approaches for supporting quality of life (Flannigan et al., 2021; Kautz‐Turnbull et al., 2022; Skorka et al., 2020). This is helpful information for clinicians to consider when developing feedback and report documents and could be considered as a key recommendation in FASD diagnostic guidelines.

The review found that receiving a FASD diagnosis produced mixed emotions, with various examples of individuals with FASD and their caregivers feeling both relieved and reassured by the diagnosis, and expressing feelings of shame, guilt, and grief. Responses ranged from solution‐focused pragmatism (being now able to plan, adjust expectations, or begin again), to experiences of depression and hopelessness. The nature of the caregiving relationship was an important factor that differentiated emotional experiences; in addition to the guilt and grief experienced by all groups at mishandling previous behaviors and acknowledging future lifelong challenges. Biological caregivers also described shame related to PAE and concerns about their future relationship with their child. The consequences of a diagnosis and variability in experiences according to different perspectives have been documented across a wide range of physical, developmental, and psychological conditions (Sims et al., 2021). This supports the value of family‐centered care as a way of working in partnership with families to better understand their circumstances and provide an individualized approach to assessment, diagnosis, and ongoing care.

After assessment, several service and family‐level barriers in accessing support were experienced. There were persistent requests for better access to interventions and therapeutic services, and for health professionals to be better‐informed about FASD. The evidence‐base is small regarding specific interventions for FASD, and while several evidence‐based interventions have been developed (Ordenewitz et al., 2021), these are yet to be widely implemented into clinical practice. Further, the evidence is currently limited to school‐aged children, with few specific interventions available for adolescents and adults (Reid et al., 2015). However, increasing knowledge of FASD across health professions has the potential to lead to more appropriate delivery of usual care for the developmental, behavioral and mental health challenges that individuals with FASD experience (Reid, Crawford, et al., 2022).

The diagnostic assessment process and the next steps arising from diagnosis had different cultural implications, with the experiences of Aboriginal Australian caregivers receiving specific attention. It is critical to note that alcohol use in pregnancy among Aboriginal Australians can only be understood in the context of colonization and its entrenched legacies within the systems of Australia. There were discussions on intergenerational trauma and shame that were triggered by receiving a diagnosis of FASD and may have significant implications for Aboriginal Australians taking up support after a diagnosis. Difficulties in engaging with Western medical terminologies within diagnostic reports and the conceptualisation of ongoing support needs being met within family networks and community were also described. These different experiences highlight the importance of providing culturally responsive assessment and diagnostic services that recognize the role of history and respect the unique experiences and cultural needs of Aboriginal families. Hamilton et al. (2020) noted, for example, that Aboriginal Australian caregivers have a demonstrated preference for visual approaches for transferring knowledge in a meaningful way, providing valuable information to inform resources.

### Strengths and limitations

This systematic review appraised studies on the lived experiences of the FASD assessment and diagnostic process. Overall, the studies reported clear aims, data collection, and analysis methods, although, in most studies, there was limited detail on participant‐researcher relationships. The application of GRADE CERQual gives a degree of confidence that the review findings were reasonable representations of the phenomenon of interest. This strengthens the potential for incorporation into evidence‐based decision making related to the provision of care. Of note, review findings with moderate confidence primarily reflected concerns about methodology and adequacy of data, highlighting a need for further research.

Review findings were constrained to the data provided by the interviews and participants within the published studies. Of note, only three studies specifically examined experiences of the FASD assessment and diagnostic process, while the other seven studies reported on these experiences as part of a larger aim to examine experiences of living with FASD. This was reflected in the low–moderate confidence ratings for the three themes relating to the diagnostic assessment process.

There was limited geographical representation with most included studies conducted in Australia and Canada, highlighting the need for further international research on individual and caregiver perspectives of the diagnostic assessment process. Few studies sought the perspective of biological caregivers, Aboriginal caregivers, and adult clients, with no studies examining the perspectives of children/adolescents who undertook an assessment for FASD. Listening to the lived experience perspectives of all stakeholders navigating this diagnostic journey is important as they have the right to be involved in decisions about their health care by having input into an evidence‐based research process (Reid, Beland, et al., 2022).

## CONCLUSIONS

Lived experience perspectives critically inform the design and development of clinical practice guidelines. This particularly applies to the assessment and diagnosis of FASD, where the evidence base provides important implications for managing referral pathways, ensuring client‐ and family‐centered assessment processes, and implementing postdiagnosis intervention and support. The current review characterized four major topics we recommend be considered in any FASD clinical process to ensure that caregivers and people with FASD are appropriately engaged and supported. Specifically, it is important that health providers identify any pre‐assessment concerns and challenges, consider a range of elements of the diagnostic assessment process, including the benefits and challenges of receiving a diagnosis, and engage in post‐assessment planning and adaptions to ensure the best quality care for people with FASD and their families.

## FUNDING INFORMATION

This research was funded by the Australian Government Department of Health Drug and Alcohol Program (GO2647). EJE is supported by an Australian Medical Research Future Fund Next Generation Fellowship (1135959). MJG was supported by a Medical Research Future Fund Translating Research into Practice (TRIP) Fellowship (1167986). PM is supported by a NHMRC Investigator Grant (1172870). DH is supported by a National Health and Medical Research Council Investigator Grant (APP1197488). AF‐J is supported by a Healthway WA Senior Research Fellowship.

## CONFLICT OF INTEREST STATEMENT

The authors have no conflicts of interest to declare.

## Supporting information


Table S1.

## Endnotes

^1^
Sanders and Buck (2010, p. 312).

^2^
Watson et al. (2013, p. 108).

^3^
Chamberlain et al. (2017, p. 103).

^4^
Hamilton et al. (2020, p. 1588).

^5^
Temple et al. (2021, p. 6).

^6^
Watson et al. (2013, p. 109).

^7^
Chamberlain et al. (2017, p. 102).

^8^
Petrenko et al. (2014, p. 1499).
