# Supplementary material for: Lived experiences of the diagnostic assessment process for fetal alcohol spectrum disorder: A systematic review of qualitative evidence
Source: Alcohol Clin Exp Res (Hoboken). 2023 May 12;47(7):1209–23. doi: 10.1111/acer.15097 (PMC10947124; doi:10.1111/acer.15097)
Supplement: Supplementary file 1 — Table S1. [file ACER-47-1209-s001.docx]

Supplemental Table 1. Summary of review findings and CERQual assessment of confidence in the evidence.

| **Review Theme** | **Studies contributing to the review findings** | **Methodological limitations** | **Relevance** | **Coherence** | **Adequacy** | **Overall Confidence in the Evidence** | **Explanation of Judgement** |
| --- | --- | --- | --- | --- | --- | --- | --- |
| **Pre-Assessment Concerns and Challenges** | | | | | | | |
| Caregiver recognition and help-seeking for child’s challenges | 3 studies  (Chamberlain et al., 2017; Salmon, 2008; Sanders & Buck, 2010) | **Minor concerns**  All 3 studies had partial or no information on researcher reflexivity; 1 study had partial ethics considerations; 2 studies had partial/unclear data collection methods | **No concerns**  1 study from Australia, 1 study from New Zealand, 1 study from Canada  Perspectives from range of caregivers were represented including biological, adoptive, foster and legal guardians.  Individuals diagnosed with FASD ranged in age from 5-30yrs. | **Minor concerns**  Within studies: clear and cogent findings  Across studies: minor inconsistency across studies – each described different behaviours and different ages of children. | **Minor concerns**  Richness: limited data to gain understanding of phenomena described (1 study was a participant quote only; 1 study didn’t provide any participant quotes)  Quantity: 3 studies contributed, sample sizes ranged from 8-11, with total n of 29 | **Moderate** | Rated down one level due to minor concerns related to methodology limitations, minor concerns about coherence of findings across studies, and minor concerns related to adequacy due to limited data and small sample size |
| Dismissal of caregiver’s concerns by health professionals | 3 studies  (Chamberlain et al., 2017; Salmon, 2008; Sanders & Buck, 2010) | **Minor concerns**  All 3 studies had partial or no information on researcher reflexivity; 1 study had partial ethics considerations; 2 studies had partial/unclear data collection methods | **No concerns**  1 study from Australia, 1 study from New Zealand, 1 study from Canada  Perspectives from range of caregivers were represented including biological, adoptive, foster and legal guardians.  Individuals diagnosed with FASD ranged in age from 5-30yrs. | **No concerns**  Clear and cogent findings within and across studies | **Very minor concerns**  Richness: sufficient data to gain understanding of phenomena described.  Quantity: 3 studies contributed, sample sizes ranged from 8-11, with total n of 29 | **High** | Did not rate down. Minor concerns related to methodological limitations, although no concerns related to relevance, coherence or adequacy. |
| FASD not considered or acknowledged | 7 studies  (Doak et al., 2019; Duquette & Stodel, 2005; Petrenko et al., 2014; Salmon, 2008; Sanders & Buck, 2010; Thomas & Mukherjee, 2019; Watson et al., 2013) | **Minor concerns**  All 7 studies had partial or no information on researcher reflexivity; 2 studies had partial/unclear ethics considerations; 2 studies had partial/unclear data collection methods; 2 studies had unclear data analysis | **No concerns**  1 study from Australia, 3 studies from Canada, 1 study each from New Zealand, U.S. and U.K.  Perspectives from a range of caregivers were represented (22 biological, 33 adoptive, 3 foster, 5 grandparents, 2 case/youth workers)^1^  Individuals diagnosed with FASD ranged in age from 3-36yrs | **No concerns**  Clear and cogent findings within and across studies | **No concerns**  Richness: sufficient data to gain understanding of phenomena described.  Quantity: 7 studies contributed, number of participants ranged from 5-31, total n of 97 | **High** | Did not rate down. Minor concerns related to methodological limitations, although no concerns related to relevance, coherence or adequacy. |
| **Diagnostic Assessment Process** | | | | | | | |
| Limited availability of diagnostic assessment services | 3 studies (Petrenko et al., 2014; Thomas & Mukherjee, 2019; Watson et al., 2013) | **Minor concerns**  All 3 studies had no information on research reflexivity; 1 study had partial information on data analysis | **No concerns**  1 study each from Canada, U.S. and U.K.  Perspectives from a range of caregivers were represented (6 biological mothers, 24 adoptive/foster parents)^1^  Individuals diagnosed with FASD ranged in age from 3-36 years) | **Minor concerns**  Within studies: clear and cogent findings  Across studies: minor inconsistency across studies – each described different behaviours and different ages of children. | **Minor concerns**  Richness: limited data to gain understanding of phenomena described  Quantity: 3 studies contributed, number of participants ranged from 5 to 31, total n of 61 | **Moderate** | Rated down one level due to minor concerns related to methodology limitations, minor concerns about coherence of findings across studies, and minor concerns related to adequacy due to limited data. |
| A safe and supportive environment without judgement is validating and empowering | 2 studies  (Chamberlain et al., 2017; Doak et al., 2019) | **Very minor concerns**  Both studies had partial information on researcher reflexivity | **Minor concerns**  2 studies from Australia  (limited geographic spread)  Perspectives from a range of caregivers and were represented (4 biological, 1 adoptive, 4 foster, 5 legal guardians, 3 grandparents)  Individuals with FASD ranged in age from 3-13yrs | **No concerns**  Clear and cogent findings within and across studies | **Minor concerns**  Richness: data to gain understanding of phenomena described.  Quantity: 2 studies contributed, number of participants ranged from 7-10, total n of 17 | **Moderate** | Rated down one level due to very minor concerns related to methodological limitations, and minor concerns related to relevance of the samples and adequacy of the data, although no concerns related to coherence. |
| Strengths-based diagnostic reports are a valuable resource | 3 studies  (Chamberlain et al., 2017; Doak et al., 2019; Hamilton et al., 2020) | **Very minor concerns**  All 3 studies had only partial information on researcher reflexivity | **Very minor concerns**  3 studies from Australia  (limited geographic spread)  Perspectives from a range of caregivers were represented (10 biological, 1 adoptive, 4 foster, 5 legal guardians, and 8 grandparents)  Individuals with FASD ranged in age from 3-17yrs | **No concerns**  Clear and cogent findings within and across studies | **No concerns**  Richness: sufficient data to gain understanding of phenomena described.  Quantity: 3 studies contributed, number of participants ranged from 7-15, total n of 32 | **High** | Did not rate down. Very minor concerns related to methodological limitations and relevance, although no concerns related to coherence or adequacy. |
| **Receiving the Diagnosis** | | | | | | | |
| Mixed emotions and improved insight | 8 studies  (Chamberlain et al., 2017; Doak et al., 2019; Duquette & Stodel, 2005; Hamilton et al., 2020; Sanders & Buck, 2010; Temple et al., 2020; Thomas & Mukherjee, 2019; Watson et al., 2013) | **Minor concerns**  All 8 studies had partial or no information on researcher reflexivity; 2 studies had unclear ethics considerations; 1 study had unclear data collection methods and 3 studies had partial or unclear data analysis | **No concerns**  3 studies from Australia, 4 studies from Canada; 1 study from U.K.  Perspectives from a range of caregivers and individuals diagnosed with FASD were represented (18 biological, 19 adoptive, 5 foster, 5 legal guardians, 8 grandparents, 20 adult individuals)^1^  Individuals with FASD ranged in age from 1-45yrs | **No concerns**  Clear and cogent findings within and across studies | **No concerns**  Richness: sufficient data to gain understanding of the phenomena described.  8 studies contributed, number of participants ranged from 7-31, total n of 105 | **High** | Did not rate down. Minor concerns related to methodological limitations, although no concerns related to relevance, coherence or adequacy. |
| Means to receive appropriate and tailored support | 6 studies  (Chamberlain et al., 2017; Doak et al., 2019; Duquette & Stodel, 2005; Hamilton et al., 2020; Temple et al., 2020; Watson et al., 2013) | **Minor concerns**  All 6 studies had partial or no information on researcher reflexivity; 1 study had unclear ethics considerations; 2 studies had unclear data analysis | **No concerns**  3 studies from Australia, 3 studies from Canada  Perspectives from a range of caregivers and individuals diagnosed with FASD were represented (11 biological, 14 adoptive, 6 foster, 5 legal guardians, 10 grandparents, and 20 adult individuals)^1^  Individuals with FASD ranged in age from 3-45yrs | **No concerns**  Clear and cogent findings within and across studies | **No concerns**  Richness: sufficient data to gain understanding of the phenomena described.  Quantity: 6 studies contributed, sample sizes ranged from 7-31, total n of 104 | **High** | Did not rate down. Minor concerns related to methodological limitations, although no concerns related to relevance, coherence or adequacy. |
| **Post-Assessment Adaptions and Needs** | | | | | | | |
| Aspirations and apprehensions about the future | 2 studies (Chamberlain et al., 2017; Doak et al., 2019) | **Very minor concerns**  Both studies had partial information on researcher reflexivity | **Minor concerns**  2 studies from Australia  (limited geographic spread)  Perspectives from a range of caregivers and were represented (4 biological, 1 adoptive, 4 foster, 5 legal guardians, 3 grandparents)  Individuals with FASD ranged in age from 3-13yrs | **No concerns**  Clear and cogent findings within and across studies | **Minor concerns**  Richness: data to gain understanding of phenomena described.  Quantity: 2 studies contributed, number of participants ranged from 7-10, total n of 17 | **Moderate** | Rated down one level due to very minor concerns related to methodological limitations, minor concerns about relevance and the geographical representation, and minor concerns about adequacy of the data. No concerns related to coherence. |
| Accessing supports and services | 5 studies (Chamberlain et al., 2017; Doak et al., 2019; Hamilton et al., 2020; Petrenko et al., 2014; Watson et al., 2013) | **Very minor concerns**  All 5 studies had only partial information on researcher reflexivity | **No concerns**  3 studies from Australia, 1 study each from U.S. and Canada  Perspectives from a range of caregivers were represented (12 biological, 27 adoptive, 6 foster, 5 legal guardians, 10 grandparents)^1^  Individuals with FASD ranged in age from 1-36yrs | **Minor concerns**  Within studies: clear and cogent findings  Across studies: minor inconsistency across studies –  reasons for lack of support varied across studies | **No concerns**  Richness: sufficient data to gain understanding of phenomena described.  Quantity: 5 studies contributed, sample sizes ranged from 7-31, total n of 98 | **High** | Did not rate down. Very minor concerns related to methodological limitations and minor concerns related to coherence of findings across studies, although no concerns related to relevance or adequacy. |

Notes: Ratings for methodological limitations, relevancy, coherence and adequacy are no/very minor, minor, moderate, serious. Ratings for overall CERQual assessment are High, Moderate, Low, Very Low confidence. ^1^Watson et al., 2013 did not provide n's for each caregiver type.
